# Paraphyly of organelle DNAs in *Cycas *Sect. *Asiorientale*s due to ancient ancestral polymorphisms

**DOI:** 10.1186/1471-2148-9-161

**Published:** 2009-07-10

**Authors:** Yu-Chung Chiang, Kuo-Hsiang Hung, Shann-Jye Moore, Xue-Jun Ge, Shong Huang, Tsai-Wen Hsu, Barbara A Schaal, TY Chiang

**Affiliations:** 1 Department of Life Sciences, Pingtung University of Science and Technology, Pingtung, Taiwan, 912, Republic of China; 2 Graduate Institute of Bioresources, Pingtung University of Science and Technology, Pingtung, Taiwan, 912, Republic of China; 3 Department of Life Science, Taiwan Normal University, Taipei, Taiwan, Republic of China; 4 South China Botanical Garden, Chinese Academy of Sciences, Guangzhou 510650, PR China; 5 Endemic Species Research Institute, Nantou, Taiwan, Republic of China; 6 Department of Biology, Washington University, St Louis, MO 63130, USA; 7 Department of Life Sciences, Cheng-Kung University, Tainan, Taiwan, 701, Republic of China

## Abstract

**Background:**

This study addresses the apportionment of genetic diversity between *Cycas revoluta *and *C. taitungensis*, species that constitute the section *Asiorientales *and represent a unique, basal lineage of the Laurasian genus *Cycas*. Fossil evidence indicates divergence of the section from the rest of *Cycas *at least 30 million years ago. Geographically, *C. taitungensis *is limited to Taiwan whereas *C. revoluta *is found in the Ryukyu Archipelago and on mainland China.

**Results:**

The phylogenies of ribosomal ITS region of mtDNA and the intergenic spacer between *atp*B and *rbc*L genes of cpDNA were reconstructed. Phylogenetic analyses revealed paraphyly of both loci in the two species and also in the section *Asiorientales*. The lack of reciprocal monophyly between these long isolated sections is likely due to persistent shared ancestral polymorphisms. Molecular dating estimated that mt- and cp DNA lineages coalesced to the most recent common ancestors (TMRCA) about 327 (mt) and 204 MYA (cp), corresponding with the divergence of cycad sections in the Mesozoic.

**Conclusion:**

Fates of newly derived mutations of cycads follow Klopfstein et al.'s surfing model where the majority of new mutations do not spread geographically and remain at low frequencies or are eventually lost by genetic drift. Only successful 'surfing mutations' reach very high frequencies and occupy a large portion of a species range. These mutations exist as dominant cytotypes across populations and species. Geographical subdivision is lacking in both species, even though recurrent gene flow by both pollen and seed is severely limited. In total, the contrasting levels between historical and ongoing gene flow, large population sizes, a long lifespan, and slow mutation rates in both organelle DNAs have all likely contributed to the unusually long duration of paraphyly in cycads.

## Background

The analysis of geographical structure and phylogeographical pattern has been a major focus in population genetics and molecular ecology [1]. Populations descending from a common ancestral population are expected to differentiate from each other and eventually show reciprocal monophyly. Reciprocal monophyly is a result of the accumulated effects of genetic drift and selection when little gene exchange occurs after divergence. In contrast, populations may be genetically similar due to recent isolation, continuous gene exchange, or secondary contact [2]. Plant species on islands provide ideal subjects for teasing apart the contrasting effects of geographical isolation and gene flow on population differentiation. Island populations are often genetically isolated from each other and most island populations are small, thus facilitating random genetic drift.

Coalescence studies provide information on the genealogical processes (backwards in time) that have led to the current apportionment of genetic variation within populations or species. Coalescence-based phylogeography uses haplotype networks, which trace the mutational relationships among alleles within or among species in a geographical context [3], to infer processes such as migration, divergence, and isolation [4-6]. Noncoding spacer regions of organelle DNAs are often used as genetic markers because they are nearly neutral with low functional constraints and are free of the complexities associated with recombination for nuclear markers [6].

Cycads are a unique and ancient lineage of land plants that have attracted much attention from evolutionary biologists [7]. *Cycas revoluta *Thunb. and *C. taitungensis *Shen *et al*. constitute the section *Asiorientales *which is a basal lineage of this Laurasian genus [8]. *Cycas revoluta *occurs in a scattered distribution pattern from the southernmost part of Kyushu and throughout the Ryukyu Islands in Japan, and is also distributed in Fukien Province of China [9]; while *C. taitungensis *is the only extant and endemic species with two endangered populations along the eastern coast of Taiwan. The two species resemble each other morphologically; a few characters such as revolute vs. plane leaflet margins distinguish the species from each other. An Eocene fossil, *C. fujiana *Yokoyama, is much like *C. revoluta*; both the morphological similarity and the occurrence of both taxa in Japan suggest that these species are the same [10]. The divergence of the species in section *Asiorientales *is thought to have occurred some 30 million years before present [10]. *Asiorientales *is allopatric from other cycads.

Both species are agriculturally important. Each year thousands of seeds and plants have been exported for use in the nursery trade and in floral arrangements [11]. In the early 20^th ^Century, cycad seeds and stems were used for making cake and soup. Cycad flour is toxic and causes neurological disorder [12]. Fermented seeds are used for producing alcoholic drinks [13]. Unfortunately, human overexploitation has led to the extinction of populations of *C. revoluta *in Fukien Province of China [9], and only two remaining populations of *C. taitungensis *occur in Taiwan [14,15]. Large specimens of *C. revoluta *are prized by the nursery industry and natural populations of *C. revoluta *in mainland China have been decimated by poachers [9]. In contrast, populations of *C. revoluta *are numerous across the Ryukyu Archipelago and Kyushu [13,16]. On the Island Amami-O-Shima, protected cycad forests contain thousands of plants [17]. Despite the high morphological similarity, the two species differ in habitat preference. *Cycas revoluta *usually grows along coasts, often subject to salt spray. In contrast, *C. taitungensis *grows on cliffs along riverbanks [18].

Island systems have long served as natural laboratories for evolutionary studies [e.g. [19,20]]. Despite their often small geographic size, islands can contain a diversity of habitats suitable for the colonization of an array of plants and animals. Once an island is colonized, population differentiation between islands can be facilitated by isolation, vicariance events and local extinction-recolonization [21]. Taiwan and the Ryukyu islands lie at the boundary between the Eurasian and Pacific plates and are a constituent of the island-arc system along the western edge of the Pacific Ocean [22]. In contrast to sequentially emerging oceanic islands of volcanic origin, these continental islands emerged almost simultaneously via collision between the continental and oceanic tectonic plates and are geologically young, estimated at 5–6 million years old [23,24]. Since emerging from the Pacific Ocean, these islands gradually acquired their floras and faunas from the Eurasian mainland [25,26]. Most island species/populations thus have close phylogenetic links to their mainland relatives.

Today, islands of the Ryukyu archipelago are isolated from each other. In the past, sea level oscillated during periodic glacial cycles (Milankovitch cycles) [27-29]. During glacial maxima, the global sea level dropped by some 100–120 meters, exposing land bridges that connected islands [30] and thus providing migration corridors between today's isolated islands. Repeated stepwise migrations, plus occasional, long-distance colonization [cf. [31-33]], would allow various plants to fill newly available habitats on islands left by the expanding or retreating glaciers. The glacial cycles of the Quaternary played an important role in the evolution of species [cf. [34,35]].

In the present study, phylogenies of the rITS region of mtDNA and the intergenic spacer between *atp*B and *rbc*L genes of cpDNA were used to trace phylogeographical relationships in *C. revoluta *and *C. taitungensis*. Organelle DNAs are maternally inherited in cycads; they disperse only via seeds [cf. [15]]. Most cycad seeds, including those of *C. revoluta *and *C. taitungensis*, are heavy and sink reducing the possibility of water/current dispersal over long distances [36,37]. The fleshy outer seed coat of cycads attracts animals, mainly rodents, small fruit-eating bats, and birds which serve as dispersal agents [38]. In natural habitats, seeds drop and occasionally disperse for a limited distance via gravity. Migration between islands is thought to be very limited and such restricted dispersal has been documented in other island species [20,39]. Ocean straits between large geographical regions (Fukien, Taiwan, and Ryukyu), and channels between islands make migration between islands unlikely in these cycads. Thus, genetic differentiation between populations for maternally inherited markers would be expected. Nevertheless, multiple colonization and recent isolation can counter forces leading to genetic differentiation between islands and monophyly [20]. Many plants distributed on the island arcs of the western Pacific Rim, including *Cunninghamia *[40], *Michelia *[41], *Kandelia *[42], *Trema *[43] and *Lithocarpus *[32,44], show little differentiation. Coalescence in island populations can be a complicated process, determined by counteracting effects of isolation (i.e., historical connection), gene flow, drift, selection and repeated colonization.

Given the similar systems of mating and demography of both cycad species, non-coding regions of organelle DNA's should provide information on species/population histories and demographic processes as well as gene flow and colonization. In this study, we examine the genetic diversity within and between species and populations, determine geographical associations and analyze phylogenetic relationships to infer the historical processes that have lead to the current pattern of genetic structure. Several specific questions are addressed:

1. Given the long time that these species have been isolated, has reciprocal monophyly of sections and species been attained?

2. Are populations and geographical regions genetically differentiated?

3. Is the spatial apportionment of genetic variation consistent with a stepwise model of colonization?

4. Is erosion of genetic diversity associated with population decline? Are levels of genetic diversity in populations of Fukien and Taiwan lower than those of Ryukyu?

## Results

### Genetic diversity within Cycas revoluta and C. taitungensis

The rITS region of mtDNA varies in length from 411 to 547 bp with a consensus length of 598 bp. The sequence length of *atp*B-*rbc*L intergenic spacer of cpDNA varies from 762 to 830 bp with a 913 bp consensus length. In total, 105 and 97 haplotypes of cpDNA as well as 170 and 55 haplotypes of mtDNA were detected in populations of *Cycas revoluta *(307 individuals) and *C. taitungensis *(102 individuals), respectively (Table 1). Haplotype sequences of mt- and cpDNAs were deposited in the GenBank database with accession numbers of FN397910-FN397954 and FM999745-FM999775, respectively.

**Table 1:** Population localities, and number of samples (N) used for a phylogeographical analysis of Cycas revoluta and C. taitungensis in Ryukyu, Fukien, and Taiwan.

Population	Area	Sampling size (N)	Symbol	Coordinate
**INGROUPS:**				
*Cycas revoluta*				
Japan: Kagoshima	Kyushu, Japan	37	KO	31°05' N, 130°70' E
Amami-O-Shima	Ryukyu, Japan	124	AM	26°90' N, 128°30' E
Okinawa	Ryukyu, Japan	26	OK	28°15' N, 130°40' E
Iriomote	Ryukyu, Japan	36	IR	24°19' N, 123°54' E
Ishigaki	Ryukyu, Japan	43	IS	24°50' N, 123°30' E
Yonaguni	Ryukyu, Japan	15	YO	24°45' N, 123°05' E
China: Fukien	Fukien, China*	26	FU	24°26' N, 118°04' E
*Cycas taitungensis*				
Taiwan: Red Leaf Conservation Area	Taitung, Taiwan	81	RL	22°85' N, 120°95' E
Coastal Conservation Area	Taitung, Taiwan	21	C	23°05' N, 121°30' E
**OUTGROUPS:**				
*Cycas taiwaniana*	Fukien, China**	1	CT	23°15' N, 113°35' E
*Cycas thouarsii*	Pingtung, Taiwan***	1	CK	21°95' N, 120°85' E

* Sampled from the South China Botanic Garden, transplanted from the Fukien wild population before extinction, which located in the Lienchiahg County, Fukien.

** Sampled from the South China Botanic Garden, transplanted from Fukien, China.

*** Sampled from the Taiwan Forestry Research Institute, transplanted form Pingtung, Taiwan.

**Figure 1:**
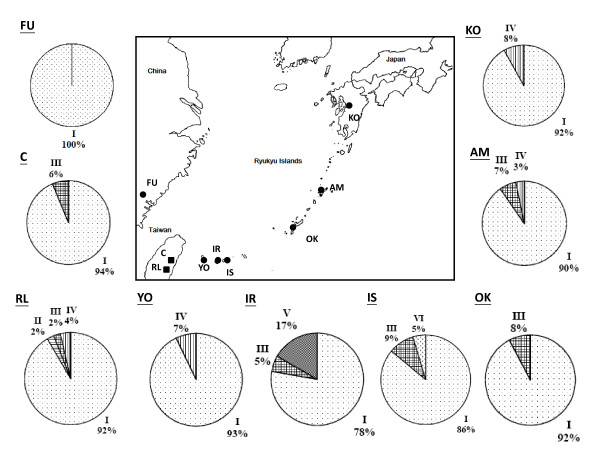
**Map showing the spatial distribution of genetic polymorphisms of mtDNA in populations of *Cycas revoluta *(circles), and *C. taitungensis *(squares)**.

Higher levels of genetic diversity of mtDNA are detected in *C. revoluta*, φ = 0.0500, than in *C. taitungensis*, φ = 0.0038 (Table 2). Among the Ryukyu Islands, populations from Yonaguni and Ishigaki have the highest levels of mtDNA diversity while the diversity in populations on Okinawa and Amami-O-Shima is relatively low. Despite its extinct status in the wild, the Fukien sample contains much higher levels of mtDNA diversity than any of the Ryukyu populations. The existence of the phylogenetically most distant mitotype, G, that occurs only in the Fukien population contributes to the high level of genetic diversity (Fig. 1). In *C. taitungensis *the level of genetic diversity is mostly associated with population size. Genetic diversity of the Red-Leaf (RL) population is about twice that of the Coastal (C) population. In contrast, the levels of genetic diversity of cpDNA are much lower in *C. revoluta*, φ = 0.008, than in *C. taitungensis*, φ = 0.018 (Table 3). Fukien has the lowest level of diversity among all populations, while the populations of Ishigaki (φ = 0.055) and Iriomote (φ = 0.022) have higher levels of genetic diversity.

**Table 2:** Haplotype diversity (h), the number of segregating sites (S), the average number of pairwise difference (k), and nucleotide diversity (π), and neutrality tests at mtDNA within cycad populations

	*h*	S	k	π	Fu and Li's D*	Tajima's D	Fu's Fs
*C. revoluta*:							
Overall:	0.932	240	11.9	0.0500 ± 0.00600			
Japan: AM	0.923	340	22.4	0.0392 ± 0.00382	-4.6531**	-2.4548***	-58.223 ***
IR	0.962	199	24.3	0.0423 ± 0.00533	-3.2490	-2.0509*	-5.908 ***
IS	0.908	399	43.3	0.0876 ± 0.02082	-3.9556**	-2.4527***	-0.656
KO	0.941	209	24.7	0.0429 ± 0.00523	-2.9419*	-2.0702*	-4.241***
OK	0.886	110	19.4	0.0335 ± 0.00437	-1.6804	-1.4166	-0.225
YO	0.933	211	56.2	0.1050 ± 0.02041	-1.0720	-1.0007	0.462
China: FU	0.982	440	108.3	0.2597 ± 0.04094	-1.6974	-1.0465	-0.326
*C. taitungensis*:							
Overall:	0.978	293	21.8	0.0383 ± 0.00470			
Taiwan: C	0.967	64	9.9	0.0197 ± 0.00598	-0.4979	-1.6527	-1.482
RL	0.975	271	23.8	0.0419 ± 0.00553	-0.0325	-2.1674*	-13.788***

*P < 0.05, **P < 0.02; ***P < 0.01

**Table 3:** Haplotype diversity (h), the number of segregating sites (S), the average number of pairwise difference (k), nucleotide diversity (π), and neutrality tests at cpDNA within cycad populations

	*h*	S	k	π	Fu and Li's D*	Tajima's D	Fu's Fs
*C. revoluta*:							
Overall:	0.959	245	5.2	0.0581 ± 0.00316			
Japan: AM	0.912	165	13.4	0.0154 ± 0.00328	-4.5405**	-1.8676*	-41.080***
IR	0.955	105	19	0.0216 ± 0.00520	-0.1257	-1.0012	-1.879
IS	0.833	328	42	0.0551 ± 0.01876	-1.7192**	-1.7929***	4.265**
KO	0.951	69	9	0.0101 ± 0.00359	-0.3475	-1.6622***	-7.981***
OK	0.797	85	12.3	0.0142 ± 0.00717	-0.9808	-1.7768***	0.674
YO	0.971	59	8.9	0.0100 ± 0.00558	-2.9824**	-2.1975***	-3.635**
China: FU	0.939	17	2.3	0.0026 ± 0.00030	-2.4588***	-1.4785**	-5.673***
*C. taitungensis*:							
Overall:	0.998	336	16.5	0.0181 ± 0.00279			
Taiwan: C	0.999	127	13	0.0148 ± 0.00646	-4.0436**	-2.5904***	-16.787***
RL	0.999	307	17.4	0.0196 ± 0.00309	-5.2128**	-2.5911***	-113.774***

*P < 0.05, **P < 0.02; ***P < 0.01

### Phylogenies of organelle DNAs in C. revoluta and C. taitungensis

**Figure 2:**
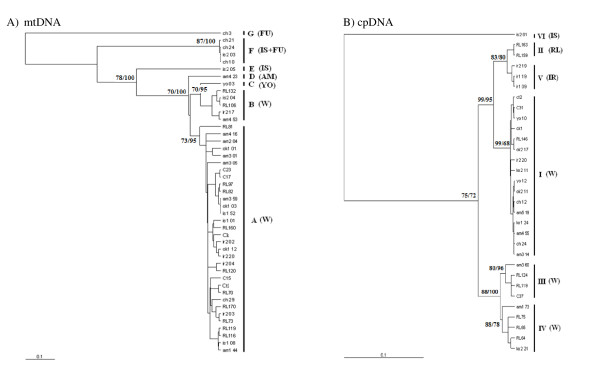
**The maximum likelihood tree of haplotypes of *Cycas revoluta *and *C. taitungensis *with outgroup sequences (O)**. Numbers at nodes indicate the bootstrap values (ML/MP) in percentage. Symbols in parentheses indicate the geographical distribution of each mitotype. W = widespread. A) mtDNA. Clades A and B occur in both species, while clades D-G are restricted to *C. revoluta*. B) cpDNA. Clades I, III and IV occur in both species, while other are restricted to *C. revoluta*.

**Figure 3:**
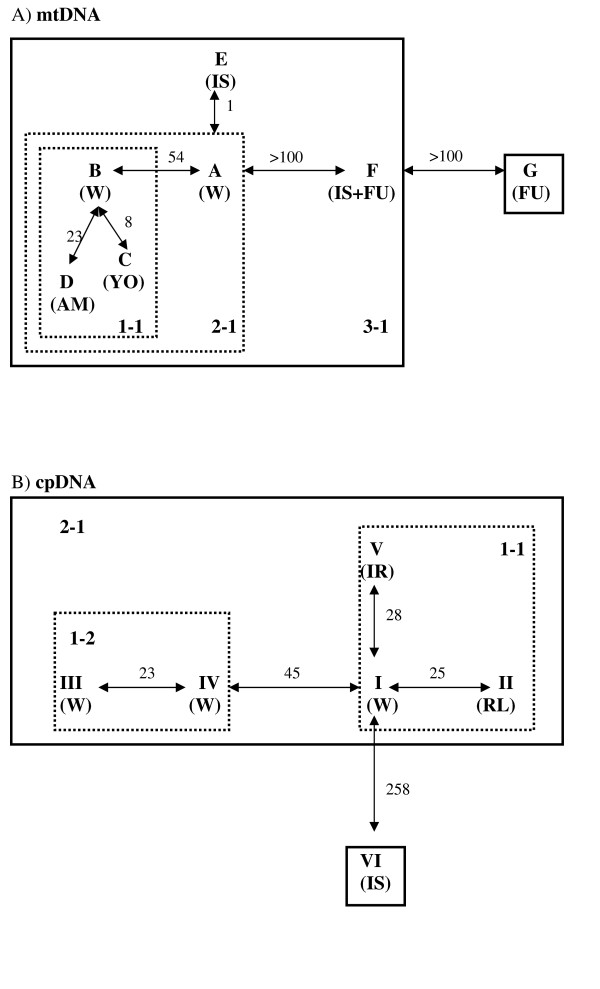
**Network of haplotypes of *Cycas revoluta *and *C. taitungensis***. Numbers at arrows indicate the mutational changes between mitotypes. A) mtDNA, B) cpDNA, C) combined data.

**Figure 4:**
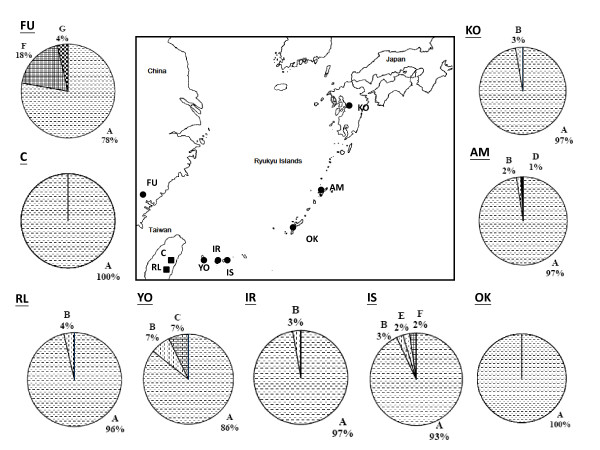
**Map showing the spatial distribution of genetic polymorphisms of cpDNA in populations of *Cycas revoluta *(circles), and *C. taitungensis *(suqares)**.

Knowledge of the process and pattern of nucleotide substitution is important for estimating the number of substitutional events between DNA sequences and phylogenetic reconstruction that incorporates models of DNA sequence evolution. Transition bias results in a substitution pattern that is characterized by transition/transversion (ti/tv) ratios typically ranging between 2 and 10 [45-47]. In contrast, higher rates of tranversional substitutions resulting in ti/tv ratios ~1 have often been equated with transitional saturation, in which the same nucleotide position undergoes multiple transitions, or considered indicative of high levels of homoplasy [48]. In this study, the transition/transversion ratios were detected to be 1.808 and 1.707 for mtDNA ITS and cpDNA, respectively, suggesting low likelihood of saturation, although relatively low. The phylogeny of the mtDNA ITS region was reconstructed by maximum-likelihood (ML) and maximum-parsimony (MP) analyses. Both analyses recover phylogenetic trees with consistent topologies. The gene tree identifies seven mitotypes, including haplotypes A to G (Fig. 2A). Reciprocal monophyly is not supported in either *Cycas *species. Geographically, mitotypes A and B are widespread (Fig. 1), while four mitotypes are restricted to single populations, including C (YO), D (AM), E (IS), and G (FU). Mitotypes C-G are absent in *C. taitungensis *while mitotype G does not occur in Ryukyus. Sequences from the two outgroups are mitotype A and do not form a monophyletic group, indicating that sections of *Cycas *are also paraphyletic for mtDNA (Fig. 2A and 3A). An ML tree based on nucleotide variation of the cpDNA *atp*B-*rbc*L intergenic spacer identifies six chlorotypes I-VI (Fig. 2B). Chlorotypes I, II, and IV are geographically widespread across islands and Fukien, while chlorotype II is restricted to *C. taitungensis*, and chlorotypes V and VI are found exclusively in Iriomote and Ishigaki (Fig. 4). The hypothesis of a molecular clock was not rejected at either locus based on the relative rate test (data not shown).

*Cycas *species are not monophyletic for cpDNA. Haplotypes of outgroups belong to chlorotype I and are also found in *C. revoluta *and *C. taitungensis *(Fig. 2B and 3B), again reflecting paraphyly of the section *Asiorientales*. In both phylogenies numerous mutations are located at tips of the network, and are derived from ancestral haplotypes at the interior nodes in the network. This pattern results in a star-like phylogeny. Furthermore, tip haplotypes are mostly confined to a single population while the interior haplotypes are geographically widespread.

### Genetic differentiation and population demography

Almost all dominant cytotypes of both organelle genes are shared among cycad populations. For mtDNA, mitotype A (95.3%) is common and widely distributed across populations, while rare mitotypes C, D, E, and G occur in single populations (Fig. 1). Likewise, for the cpDNA intergenic spacer, chlorotype I (90.1%) is the most common and widespread in all populations, while rare chlorotypes II, V, and VI are restricted to single populations (Fig. 4). Despite the lack of physical linkage between the two organelle genomes, high levels of linkage disequilibrium are detected in both *Cycas *species (P < 0.05). All rare mitotypes (4.7%), including B-G, are associated with chlorotype I; while all rare chlorotypes II–VI (9.9%) are associated with the mitotypes A, in the pooled data reflecting a nonequilibrium state.

Since common haplotypes are shared among populations, cycads show little genetic differentiation at the two organelle DNA markers. *Nm *values obtained from Fst estimates in *C. revoluta *vary among pairwise comparisons between populations (Tables 4 and 5). High levels of differentiation between Japan and China populations are detected for all comparisons, e.g., Fst of 0.115 (P < 0.01) between OK and FU based on mtDNA, and Fst = 0.159 between IR and FU (P < 0.01) based on cpDNA (Tables 4 and 5). For example the estimated number of migrants per generation is high between populations C and R of *C. taitungensis*, *Nm *= 90.41 and 23.18 based on cp- and mtDNAs, respectively (60). The *Nm *values based on Fst estimates from mtDNA average 106.10 which is greater than the average from cpDNA data, 10.95 (Tables 4 and 5).

**Table 4:** Pairwise comparisons of Fst (below diagonal) and Nm values (above diagonal) between populations of Cycas revoluta deduced from mtDNA sequences.

Fst\Nm	AM	IR	IS	KO	OK	YO	FU
AM		44.16	89.16	90.17	45.15	12.29	4.19
IR	0.011		105.02	111.27	1030.15	18.48	4.00
IS	0.006**	0.005		475.91	70.97	24.17	7.31
KO	0.006	0.005	0.001		51.75	17.8	4.13
OK	0.011*	0.001	0.007	0.010**		11.7	3.87
YO	0.039*	0.026	0.020	0.027	0.041		6.52
FU	0.107**	0.111**	0.064**	0.108**	0.115**	0.071	

Probability value associated with the Fst is shown. The Fst values significantly greater than zero are noted by adding *P < 0.05 and **P < 0.01

**Table 5:** Pairwise comparisons of Fst (below diagonal) and Nm values (above diagonal) between populations of Cycas revoluta deduced from cpDNA sequences.

Fst\Nm	AM	IR	IS	KO	OK	YO	FU
AM		6.02	13.58	17.86	7.63	3.71	7.13
IR	0.077*		38.08	3.07	7.38	3.89	2.64
IS	0.036*	0.013		8.06	12.57	8.58	6.49
KO	0.027	0.140**	0.058		3.59	2.34	10.25
OK	0.061	0.063	0.038	0.122		62.62	2.66
YO	0.119	0.114*	0.050	0.176	0.008		1.87
FU	0.066	0.159**	0.072*	0.047	0.158	0.211	

Probability value associated with the Fst is shown. The Fst values significantly greater than zero are noted by adding *P < 0.05 and **P < 0.01

The program IM defined six demographic parameters estimated by the MCMC (Markov chain Monte Carlo) multiple loci simulations and tested for an 'isolation with migration' model in cycads. Of these demographic parameters, the distribution of migration parameters reveals a peak at the lower limit of resolution in one direction from Japanese populations to the Chinese population in *C. revoluta *(Table 6). When the MLE (maximum likelihood estimates) for the migration parameters are transformed into the population migration rates, M_1 _= 2*N*_1_*m*_1 _= *m*_1_*(θ_1_/2) estimated from Japanese populations to the Chinese population in *C. revoluta *is higher than the estimate, M2 = 2N_2_m_2 _= m_2_*(θ_2_/2), from China to Japan, i.e., 8.0134 vs. 0.5431 based on the combined data (Table 6). The Neτ (the product of effective population size and the generation time in years) value is also assessed; a higher effective population size is detected for Japan's populations than China population, i.e., Neτ = 4.753*10^6 ^vs. 1.437*10^5 ^based on the combined data (Table 6).

**Table 6:** Scaled parameter estimates for IM model as inferred from the combined data of cpDNA and mtDNA.

	θ_1_^a^	θ_2_^a^	θ_A_^a^	*m*_1_^b^	*m*_2_^b^	N_1_τ^c^	N_2_τ^c^	N_A_τ^c^	2N_1_m_1_	2N_2_m_2_
A. HiPt^d^	8.9914	0.2426	10.9320	1.4500	3.5500	4.517*10^6^	1.210*10^5^	5.492*10^6^	6.5188	0.4306
95%HPDLo^e^	6.4040	0.2426	5.4983	0.6500	1.3500					
95%HPDHi^e^	51.8138	2.6683	70.9610	14.0500	7.4500					
B. HiPt^d^	10.0668	0.2860	10.3903	1.9978	4.1023	5.057*10^6^	1.426*10^5^	5.219*10^6^	10.0557	0.5867
95%HPDLo^e^	7.8028	0.2860	5.2962	0.8820	1.5548					
95%HPDHi^e^	79.2004	2.5066	36.1031	16.1243	7.8965					
C. HiPt^d^	9.3231	0.3356	10.6652	1.6345	3.6577	4.683*10^6^	1.674*10^5^	5.358*10^6^	7.6193	0.6138
95%HPDLo^e^	7.2336	0.3356	5.3365	0.7586	1.4723					
95%HPDHi^e^	68.5891	2.4523	65.2581	15.4572	7.6894					
Average	9.4604	0.2881	10.6634	1.6941	3.7700	4.753*10^6^	1.437*10^5^	5.358*10^6^	8.0134	0.5431

Note: Values are presented for each of the three runs with different seed numbers (A, B and C).

^a ^The population size parameters for the Japan population (*θ*_1_) and Fukien population (*θ*_2_) *of C. revoluta*, and ancestral population (*θ*_A_).

^b^Migration rate estimate (*m*_1 _– from Japan to Fukien population; *m*_2 _– from Fukien to Japan population).

^c ^Effective population size estimate (Nτ: population size × generation time) for Japan population (N_1_τ) and Fukien population (N_2_τ) *of C. revoluta*, and ancestral population (N_A_τ).

^d^The value of the bin with the highest count (HiPt).

^e^The lower (HPD95Lo) and upper (HPD95Hi) bound of the estimated 95% highest posterior density (HPD) interval.

**Figure 5:**
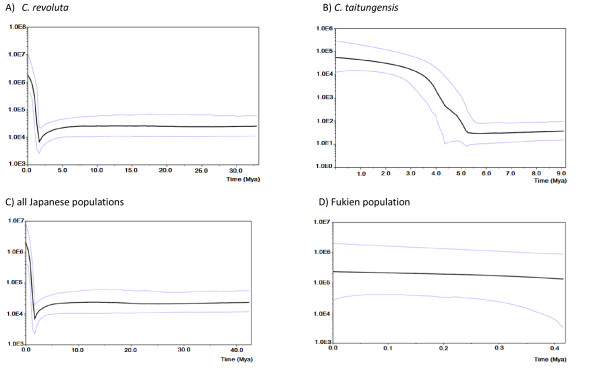
**Bayesian skyline plot based on the mtDNA for the effective population size fluctuation throughout time**. X axis: population size × generation time; Y axis: time (million years ago). A) *Cycas revoluta*; B) *C. taitungensis*; C) all Japanese populations of *C. revoluta*; D) the Fukien population of *C. revoluta *(black line, median estimations; area between gray lines, 95%

**Figure 6:**
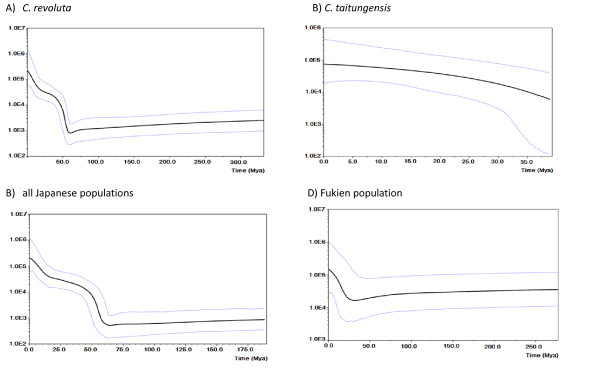
**Bayesian skyline plot based on the cpDNA for the effective population size fluctuations throughout time**. X axis: population size × generation time; Y axis: time (million years ago). A) *Cycas revoluta*; B) *C. taitungensis*; C) all Japanese populations of *C. revoluta*; D) the Fukien population of *C. revoluta *(black line, median estimations; area between gray lines, 95% confidence interval.

In the study, the demographic scenarios for the two species and populations of Japan and China of *C. revoluta *are represented by the Bayesian skyline plot (Fig. 5 and 6). Based on the pattern of variation in mtDNA, a long history of constant population size of *C. revoluta*, followed by a slight decline (bottleneck) with subsequent demographic expansion, is recovered (Fig. 5A). At the population level, in *C. revolute*, the Japanese and Fukien populations also display similar patterns (Fig. 5C and 5D). In contrast, slow population growth is detected in *C. taitungensis *based on the Bayesian skyline plot (Fig. 5B). Based on cpDNA, a scenario of population expansion is suggested for both *C. revoluta *and *C. taitungensis*, while a weak bottleneck event may have occurred in *C. revoluta *(Fig. 6A and 6B). Likewise, Japan's populations of *C. revoluta *almost remain nearly constant before a recent population expansion, whereas, the Fukien's population did not undergo large fluctuations in population size until its recent extinction (Fig. 6C and 6D).

**Figure 7:**
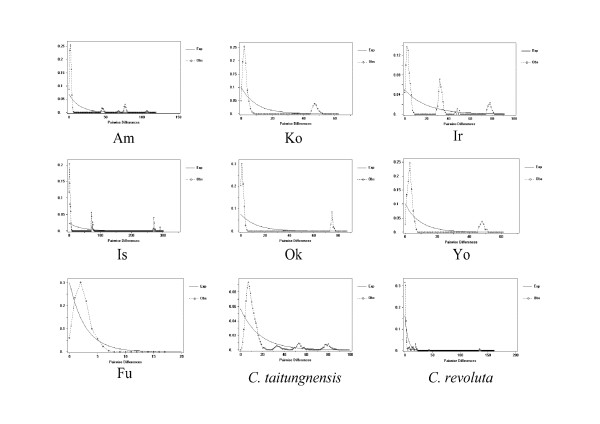
**Mismatch distributions of mtDNA haplotypes based on pairwise sequence differences against the frequencies of occurrence for cycad species and populations**.

**Figure 8:**
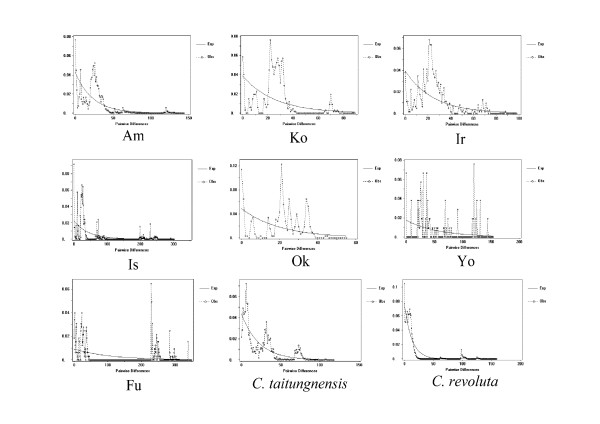
**Mismatch distributions of cpDNA haplotypes based on pairwise sequence differences against the frequencies of occurrence for cycad species and populations**.

We also use mismatch distribution analyses to infer the long-term demographic history of populations. Mismatch distribution analyses (Fig. 7 and 8) show different patterns between the cp- and mtDNA genomes. In most cycad populations, cpDNA has a unimodal mismatch distribution (Fig. 8), while multimodal and very ragged distributions are detected in mtDNA within all populations (Fig. 7). Nevertheless, when sequences from all populations are pooled together, both cp- and mtDNAs have unimodal mismatch distributions in *C. revoluta*, while only cpDNA displays a unimodal mismatch distribution in *C. taitungensis*. Unimodal mismatch distributions are often the result of past demographic expansions [50,51]; whereas, multimodal mismatch distributions usually indicate stationary population structure. Since the data used to produce mismatch distributions are not independent [52], Tajima's *D*, Fu and Li's D* and Fu's Fs statistics can be used to detect departure from population equilibrium. In this study, values of Fu and Li's D* based on cpDNA sequences for *C. revoluta *and *C. taitungensis *are significantly negative, except for populations IR, KO, and OK (Table 3); while Tajima's D statistics are all significantly negative, except for the population IR. Fu's Fs statistics are significantly negative, except for populations IS, IR, and OK (Table 3). Likewise, Fu and Li's D*, Tajima's D, and Fu's Fs statistics are significantly or marginally significantly negative based on mtDNA data in most populations (Table 2).

### Coalescence of lineages and molecular dating

**Figure 9:**
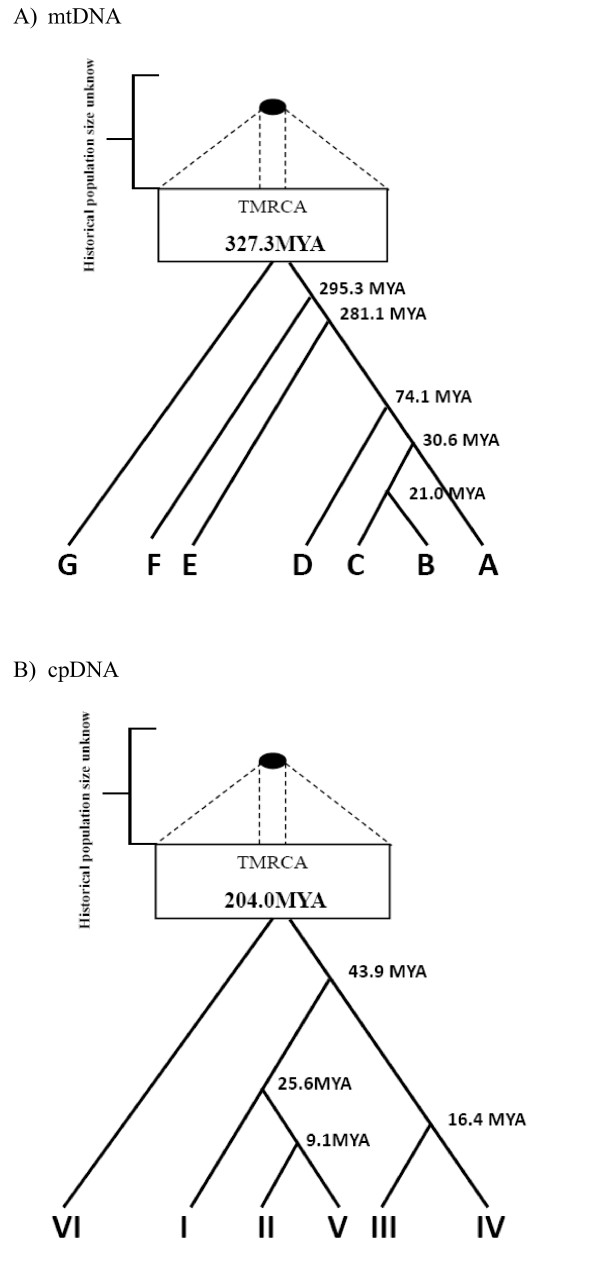
**Coalescence-based analyses of lineages**. Time coalesced to the most recent common ancestor (TMRCA) of major lineages are indicated. A) mtDNA, B) cpDNA.

Bayesian estimates of the mutation rates and the age of the most recent common ancestor (TMRCA) of the cycad sequences were obtained using BEAST v. 1.3. For mtDNA, all the haplotypes coalesced at about 327.3 MYA, while all chlorotypes could be traced back to a common ancestor about 204.0 MYA (Fig. 9). In the mtDNA haplotype tree, clusters (A, B, C) vs. D split at some 74.1 MYA; and the clusters A and (B, C) coalesced at about 30.6 MYA. Likewise, in the cpDNA tree, clusters I and (II, V) split some 25.6 MYA, while cluster (I, II, V) diverged from its sisters (III, IV) an estimated 43.9 MYA. Almost all coalescences of the major lineages predate the formation of the section *Asiorientales*. Coalescent events occurred much earlier in mtDNA than in cpDNA. On the other hand, most tip haplotypes in both gene trees coalesced recently to their common ancestors.

## Discussion

### Highly polymorphic organelle DNAs in Cycas revoluta and C. taitungensis

Genetic variation in the two cycad species is high, comparable to or higher than many tree species (such as *Quercus *[31,49]; *Fagus *[50]; *Lithocarpus *[32,44,51]. Such high levels of genetic variation within mt- and cpDNAs suggest that these are ancient lineages with enough time for mutations to accumulate within lineages, given the slow rate of organelle evolution [52]. Evidence of the deep splits in both phylogenetic trees (Fig. 2) and the estimated dates of divergence support this scenario. Moreover, star-like phylogenies with a mixture of long and short branches, which represent recently evolved haplotypes, illustrate the high levels of variation.

Although the two cycad species have high levels of genetic diversity at both loci, the loci do not vary in concert. For example, the *C. revoluta *population YO possesses the highest level of mtDNA variation while having the lowest level of cpDNA diversity. Similarly, the Fukien China *C. revoluta *population is highly diverse for mtDNA haplotypes while containing lower than average of cpDNA haplotypes. The large native cycad population on the island of Amami-O-Shima (AM) has genetic diversity at both loci comparable to or even lower than most populations.

In this study, genetic diversity tends to be higher in the south than in the north. A cline-like distribution of genetic diversity from south to north occurs in many continental plants of the Northern Hemisphere and is thought to be related to colonization history over glacial cycles [cf. [34,53]]. Considering the geological history of the Taiwan-Ryukyus Archipelago, one expects a similar pattern for these cycad species where colonization occurs in a stepwise manner from China northwards through the Archipelago. Genetic diversity should decrease to the north, assuming that a small number of individuals colonize each subsequent island in a stepwise manner. However, the occurrence of highly diverse organelle DNAs across the range of *Cycas revoluta *and *C. taitungensis*, and many other tree species of the Taiwan-Ryukyus Archipelago suggests that single stepwise colonization events may not be the sole determinant of the overall pattern of genetic diversity [cf. [33]].

Austerlitz *et al*. [51] suggest that the level of diversity in tree species that recolonize glaciated regions is affected by restricted gene flow but is also tempered by their demography. In tree populations, the genetic effects of founder events are reduced due to overlapping generations and delayed reproduction associated with long life spans. These are traits found in most long lived trees including these cycads, which are characterized by great longevity, overlapping generations, age structured populations and a juvenile phase of 30 to 50 years. During the juvenile period when cycads grow vegetatively, newly founded cycad potentially populations can increase in number for many years only through the arrival of new migrants, especially when migration corridors across islands for migration are available. This pattern of colonization and reproduction effectively acts to increase in the number of initial colonizers of a given population and in turn decreases any founder effects. Even given a high likelihood of stepwise colonization along the Taiwan-Ryukyu Archipelago, as observed in the *Pinus luchuensis *complex [52,33], multiple colonization events could compensate for potential reduction in genetic diversity during each founder event.

Secondary contact of ancestral lineages can be another factor that contributes to high genetic diversity. The coalescence-based analysis reveals that mitotypes F and G diverged some 327.3 and 295.3 million years ago, respectively. Today these haplotypes exist exclusively in the mainland Fukien (mitotype G), and in the Fukien and Ishigaki (mitotype F) populations. Mitotype F is common in the Fukien population, suggesting an origin most likely on the Asian mainland with subsequent dispersal northward onto Ishigaki. Such secondary contact of divergent lineages is consistent with a U-shaped site-frequency distribution according to Wakeley and Aliacar's simulations (Fig. 8) [54].

### Isolation and lack of geographical subdivision

Current gene flow between islands is greatly restricted due to their geographical isolation and the low dispersability of cycad seeds in water [36]. Thus, one expects that island populations should be significantly differentiated from each other. But, like many other tree species of the Taiwan-Ryukyu Archipelago [33], no significant geographical differentiation is detected between cycad populations among islands. Fst values are small and all pairwise *Nm *values, deduced from Fst values, are much greater than one (Tables 4 and 5). The *Nm *values based on mtDNA data are about ten-fold greater than those based on cpDNA sequences, 106.10 vs. 10.95. While both organelles are transmitted only via seeds, the difference in *Nm *values determined from cp- and mtDNAs coupled with deviation from an isolation-by-distance model, and the lack of geographical subdivision and genetic differentiation, suggest that the two loci are responding to different evolutionary forces.

Clearly, *Nm *values cannot be interpreted as current gene flow between populations. These high *Nm *values likely represent either ancient migration events or shared ancestral polymorphisms [55,56]. For example, the *Nm *values between Chinese and Japanese populations range from 3.87 to 7.31 (mtDNA) and 1.87 to 10.25 (cpDNA) (Tables 4 and 5). Testing of 'isolation with migration' (IM), nevertheless, reveals asymmetrical, historical gene flow with more migrants of *C. revoluta *from Japan to China than vice versa, 8.0134 vs. 0.5431 based on combined data. These results suggest long periods of isolation with limited genetic exchange between the two geographical regions. *C. taitungensis *shows a similar asymmetric pattern of ancient gene flow. Migration is estimated to be more frequent from population C to population RL (3.86445 vs. 41.10288, cpDNA, and 2.25430 vs. 48.99630, mtDNA), contributing to the lack of genetic differentiation between populations, as indicated by Fst values of 0.0056 (cpDNA) and 0.021 (mtDNA) [cf. [55]].

Like other island species, cycad populations were colonized via founders from neighboring islands or the mainland. Today, deep water channels hinder seed dispersal among islands and most seed dispersal occurs only within islands. However, during the glacial maxima [cf. [29]], sea levels were much lower and migration could have occurred among islands. The rise of sea levels during glacial retreats terminated between-island seed exchange and isolated cycad populations of the Ryukyu Archipelago from each other [57]. The time since isolation approximates 200–400 cycad generations. Given the very short time that populations have been isolated and the low rate of molecular evolution for both organelle loci, it is not surprising that populations show genetic coherence. A recent simulation study [51] indicates that tree populations can remain genetically similar even with an isolation-by-distance pattern of colonization and can yield a phylogeographical pattern with little differentiation, here for the cycads of the Taiwan-Ryukyu Archipelago. The high levels of nucleotide diversity in both organelle DNAs and the existence of the highest number of private cytotypes suggest that population IS of the south Ryukyu and population RL of Taiwan may have acted as glacial refugia for *C. revoluta *and *C. taitungensis*, respectively.

### Paraphyly of loci in Cycas species and sections

Although cycad populations are undifferentiated from each other due to recent historical gene flow (Table 6), given that the generic sections of cycads sections have been isolated for very long periods of time, one would expect monophyly at the sectional level. Nevertheless, both mt- and cpDNAs are paraphyletic or polyphyletic between section *Asiorientales *and its sisters. Such lack of reciprocal monophyly between taxa may result from selection, e.g., the S locus of *Brassica *[58], and α-tubulin genes of *Miscanthus *[59], lineage sorting [60], or hybridization/introgression [61]. Genetic exchange among species via hybridization and introgression introduces foreign alleles into a species or population and can result in a genetic admixture. In contrast, lineage sorting occurs when populations or species are recently diverged and retain ancestral polymorphisms [cf. [5,52]].

Hybridization and/or lineage sorting are likely causes of shared polymorphisms in *Cycas *given that these are neutral loci and thus not under strong selection. Discerning between hybridization and lineage sorting can be difficult [61-64], since most statistical analyses have limited power. However, in this study neither hypothesis can by itself explain the current patterns of genetic variation among sections. First, all four *Cycas *species are allopatric which greatly reduce the possibility of recent recurrent interspecific hybridization. Lineage sorting can result in shared polymorphism but requires a relatively short time of isolation. The fossil record indicates that the sections of *Cycas *have been diverged for 30 million years, making such a scenario unlikely [10].

The pattern of coalescing DNA segments provides information on the genealogical processes that occur within populations [4,65,66]. The star-like phylogeny of the organelle trees shows that recently derived haplotypes are numerous, external on the networks and are geographically restricted. Ancestral haplotypes are found at interior nodes, and are geographically more widespread than the recently derived tip haplotypes. This pattern is consistent with a birth-and-death model where most mutations go extinct with only very few ancestral polymorphisms surviving to become dominant [67]. If the dynamics of mutations and fixation do not follow a birth and death model, one expects topologies with deep splits for most haplotypes.

The pattern of mutations in cycads follows a 'surfing' model [68], which predicts that a majority of new mutations do not spread geographically and either remain at low frequencies or are lost by genetic drift. Only successful 'surfing mutations' can reach high frequencies and eventually occupy a large geographical region [69]. In this model both population size and migration rate determine the success of new mutations. Deme size not only affects the probability of a mutation to 'surf' but also determines its final frequency and spatial distribution. When the effective population size is small, a mutation within a population has a high probability of extinction due to genetic drift and it is able to spread only if it reaches a very high frequency by chance. In contrast, large effective population sizes counter genetic drift while a large number of exchanged migrants between neighboring demes prevents a mutation from reaching high local frequencies [68]. Accordingly, as most cycad populations were probably large (Fig. 5 and 6), one expects to observe numerous recently derived mutations that were derived from a single ancestral haplotype, and the observation that few haplotypes appear to survive over long periods (Fig. 2). Most of the new mutations appear to be ephemeral and can be lost from a population or species during population contraction (the glacial maximum). As a consequence, very few cytotypes, *e.g*., mitotypes A, and B, and chlorotypes I, and III, survived and became dominant across the cycad populations. Such a birth-and death process corresponding to demographic fluctuations may have occurred regularly in cycads over repeated glacial cycles. As a result, the existence of rare and geographically restricted cytotypes contributes to the high levels of genetic diversity within populations and within species, whereas, they played very minor roles in differentiating populations or species from each other due to their rarity.

While the surfing phenomenon explains the dominance of widespread haplotypes and low genetic differentiation between cycad populations, it does not explain shared ancestral polymorphisms between sections. Both genetic drift and changes in population size have most likely influenced polymorphism as well. Population contraction-expansion, which has occurred regularly in the Quaternary, provides an opportunity for rapid stochastic loss or fixation of alleles (haplotypes). Rapid population growth and expansion in cycads is suggested by the star-shape phylogenies found in most lineages of both organelle genomes, or the Bayesian skyline plot and by the observation that DNA sequences are saturated with singletons at both the population and species levels (Fig. 3 and 4) [cf. [70,71]], as well as the Bayesian skyline plotting. A mixture of long and short branch lengths in the star-like phylogenies could also be caused by heterogeneous mutation rates. The significant values for Tajima's D and Fu and Li's D* statistics, nevertheless, favor a demographic expansion hypothesis [72].

Mismatch distribution analyses are also used to infer the long-term demographic history of populations. In this study, contrasting patterns of unimodal vs. multimodal mismatch are found for mt- and cpDNAs (Fig. 7 and 8). Usually, unimodal mismatch distributions are interpreted as the result of past demographic expansions [73,74], whereas multimodal mismatch distributions indicate stationary population sizes. These contrasting patterns in cycads are difficult to interpret although a mismatch inconsistency between different data sets is not unprecedented. A well-known example of contrasting mismatch patterns occurs between expanding human Neolithic and hunter-gatherer populations [75]; the unimodal distribution for Neolithic populations is interpreted as the result of greater *Nm *values [76]. Even with a multimodal distribution, demographic expansion cannot be rejected, especially if the *Nm *values are small. In this study, the contrasting mismatch patterns between unlinked organelle genomes seem to be uncorrelated with *Nm *values. The ragged distributions in mtDNA are more likely attributable to the low τ (fig. 5). In cycads, a very recent population expansion about 200–400 generations, a slow evolutionary rate, and putative intramolecular recombination [data not shown; cf. [55]], may preclude mismatch distribution analyses from assessing recent population expansion. Nevertheless, Tajima's D, Fu and Li's D*, and Fu's Fs statistics provide independent estimates for spatial expansion, especially considering that the DNA sequences are from neutral noncoding spacer regions.

Coalescence-based analyses measure the time of coalescence to the most recent common ancestor (TMRCA). In both organelle DNAs, the divergence time is estimated at 327.3 MYA in mtDNA and 204.0 MYA in cpDNA, agreeing with the divergence of major sections of cycads in the Mesozoic [77]. Moreover, the split of the common haplotypes, mitotype B and chlorotype I, from their sister haplotypes occurred about 21.0 and 25.6 MYA, respectively in the Tertiary period when cycads were diversifying and when divergence of section *Asiorientales *(30 MYA) occurred. Since then the two dominant cytotypes have persisted in cycads [cf. [55]]. The sharing of common cytotypes in both genomes is consistent with range contraction-expansion during glacial cycles where common haplotypes could persist, while most rare, newly derived haplotypes were lost. Consequently, both phylogenies show topologies with few ancient coalescence events vs. numerous recent coalescence events.

The evolution of these two cycad species is complex, affected by range expansion and contraction, fluctuations in population size, widespread historical and current restricted gene flow during complex glacial cycles as well as low rates of molecular evolution in organelle DNA. All of these processes result in a pattern of genetic similarity among populations, a lack of reciprocal monophyly, and long coalescent times.

## Conclusion

In summary, paraphyly at both organelle DNAs has been long maintained in *Cycas *section *Asiorientales *ever since the split from its sisters some 30 million years ago. Large population sizes of the constituting species *C. revoluta *and *C. taitungensis *contributed to the lack of reciprocal monophyly betwen *Cycas *sections. Providing limited current seed dispersal across oceans, unexpected low levels of geographical subdivision are attributable to the sharing of common haplotypes among island populations. Demographic expansion and contraction with fluctuating population sizes, widespread historical vs. current restricted gene flow during complex glacial cycles, and low rates of molecular evolution in organelle DNA all have to be aroused to explain such population structuring.

## Methods

### Plant materials, PCR, and nucleotide sequencing

We assessed levels of sequence variation for cp and mt-DNAs among individuals and populations of *Cycas revoluta *and *C. taitungensi*s in Taiwan, Fukien, and Ryukyus (Table 1, Fig. 1 and 4). A total of 307 and 102 plants of *C. revoluta *and *C. taitungensis*, respectively, were sampled. *Cycas revoluta *is extinct from the wild in Fukien; only old trees were collected from public gardens or nurseries to avoid sampling from recently introduced material. The samples of *C. revoluta *in China were collected from South China Botanic Garden, which had plants collected from the Fukien wild population before extinction, which located in the Lienchiahg County, Fukien. An additional six native island populations were collected in Ryukyus for *C. revoluta *and two populations of *C. taitungensis*. Two species, *C. taiwaniana *Carruthers (Section *Stangerioides*) and *C. thouarsii *R. Brown (Section *Rumphiae*) [cf. [8]], were chosen as outgroups. *C. taiwaniana *was obtained from the South China Botanic Garden, transplanted from a native population in Fukien, China, and *C. thouarsii *was sampled from the Taiwan Forestry Research Institute, transplanted from a native population in Pingtung, Taiwan. Young and healthy leaves were collected in the field, rinsed with tap water and dried in silica gel. All samples were stored at -70°C until they were processed. The voucher specimens (*Hsu s.n*.) of this research are deposited in the Herbarium, Endemic Species Research Institute, Nantou, Taiwan. Leaf tissue was ground to a powder in liquid nitrogen and stored at -70°C. Genomic DNA was extracted from the powdered tissue following a CTAB procedure [78]. The *atp*B-*rbc*L noncoding spacer of cpDNA and the rDNA internal transcribed spacer (rITS) of mtDNA were amplified and sequenced. PCR amplification was carried out in 100 μL reaction using 10 ng of template DNA, 10 μL of 10× reaction buffer, 10 μL MgCl2 (25 mM), 10 μL dNTP mix (8 mM), 10 pmole of each primer, 10 μL of 10% NP-40, and 2 U of *Taq *polymerase (Promega, Madison, USA). The reaction was programmed on a MJ Thermal Cycler (PTC 100) as one cycle of denaturation at 95°C for 4 min, 30 cycles of 45 s denaturation at 92°C, 1 min 15 s annealing at 52°C, and 1 min 30 s extension at 72°C, followed by 10 min extension at 72°C. A pair of universal primers for cpDNA *atp*B-*rbc*L spacer [79] or mtDNA rITS (mito 18 s F 5'-GTG,AAG,TCG,TAA,CAA,GGT,AGC-3'; mito 5 s R 5'-TCG,AGG,TCG,GAA,TGG,GAT,CGG-3') [cf. [80]], dNTP and *Taq *polymerase were added to the above ice-cold mix. The reaction was restarted at the first annealing at 52°C.

PCR products were purified by electrophoresis in 1.0% agarose gel using 1 × TAE buffer. The gel was stained with ethidium bromide and the desired DNA band was excised and eluted using QIAquick Gel Kit (QIAGEN). For all individuals, purified amplicons were ligated to a pGEM-T easy vector system (Promega). Five clones were randomly selected and purified using the Plasmid Mini Kit (QIAGEN). Purified cloned ampicons were sequenced in both directions using BigDye chemistry (Perkin Elmer) on ABI 377A automated sequencer (Applied Biosystems). Primers for sequence determination were T7-promoter and SP6-promoter located on p-GEM-T easy Vector termination site. As no within-individual variation was detected for either DNA marker, direct sequencing with eluted PCR products was also conducted to all individuals.

### Data analysis

#### Sequence alignment and phylogenetic analyses

Nucleotide sequences were aligned with the program BlastAlign [81]. After the alignment, the indels were coded as missing data. Phylogenetic trees were reconstructed using maximum likelihood (ML) analyses of the nucleotide sequences with software PHYML v2.4.5 [82] and bootstrap consensus values calculated using 1,000 replicates. The general time reversible GTR + I + G model with 6 substitution categories was determined to be the most suitable model by Modeltest v3.6 [83] and was used for all subsequent nucleotide analyses. Maximally parsimonious (MP) trees were also sought using PAUP heuristic search strategies [84]. The length of the shortest trees was obtained by initiating 1,000 heuristic searches, each using random addition starting trees, with tree bisection-reconnection (TBR) branch swapping. The equally MP trees were then used as starting trees for TBR branch swapping. In all analyses, the maximum number of trees to be saved was set at 5000. Bootstrap values [85] were calculated from 1,000 replicate analyses using a heuristic search strategy, simple addition sequence of the taxa, and TBR branch swapping. The values of random number seed set as 64238 and 70189 for mtDNA and cpDNA data sets, respectively, were used to start the random number generator. Relationships among haplotypes were determined and displayed as nested phylogenies. In order to visualize the phylogeographical relationships, a network of each locus based on statistical parsimony was constructed with the computer program TCS [86]. A distance matrix was composed of all pairwise comparisons of haplotypes, for which the maximum number of mutations was determined that did not exceed the probability of parsimony by 0.95 [as defined in [87]]. All haplotypes satisfying this parsimony criterion were then connected to a single network, while those of which the probability exceeded 0.95 were resolved as a separate network. According to coalescent theory, tip nodes of a network are likely to represent descendents derived from ancestral, interior nodes [cf. [88,89]].

The hypothesis of a molecular clock was tested by relative rate tests [90,91]. The null hypothesis of a molecular clock suggests that the number of nucleotide substitutions between two lineages would be the same. Based on the assumption of a normal distribution of nucleotide substitutions [91], the hypothesis of a molecular clock will be rejected with 95% significance, when the difference of substitution rates between two lineages is greater than 1.96 times the standard error.

#### Population genetic analysis

Summary statistics such as the number of segregating sites (S), and the average number of pairwise difference (k) between haplotypes were determined. The level of genetic diversity within populations was quantified by measures of nucleotide divergence, θ [92] and φ [93], using DnaSP Version 4.10 [94]. In order to make inferences about demographic changes in the cycads, we employed both mismatch distributions and statistical tests of neutrality. We calculated Tajima's D [91], Fu and Li's D* statistic [95], and Fu's Fs statistics [96], which is powerful for detecting population growth [97], in the noncoding DNA fragments as indicators of demographic expansion in DnaSP. We also investigated the historical demographics of populations by plotting mismatch distributions [73] and comparing them to Poisson distributions. Mismatch distributions for each sample were used to distinguish between models that invoke past exponential growth and historical population stasis. Parameters of demographic expansion were estimated using the methods of Schneider and Excoffier [98]. The validity of the demographic expansion hypothesis was tested using a parametric bootstrap approach, in which the sum of squared deviation (SSD) between the observed distribution and the expected distribution was compared to the SSD between the simulated distributions and the expected distribution (Arlequin version 3.0 [99]). Patterns of geographical subdivision and genetic differentiation were estimated hierarchically in DnaSP. Statistical significance of Fst estimates was assessed using software Arlequin with 10,000 permutations.

#### Estimation of coalescence times of cp and mtDNA lineages

We estimated the coalescence time of the the sister lineages of *C. taitungensis *and *C. revoluta *split for both mt- and cpDNAs. To estimate divergence between lineages a well-supported rate of evolution is required. In seed plants, evolutionary rates are estimated at 1.01 × 10^-9 ^and 4.50 × 10^-10 ^substitutions per site per year for synonymous sites of cp- and mt-DNAs, respectively [cf. [52,100]]. These values approximate the evolutionary rates of introns and noncoding spacers of organelle DNAs. Bayesian estimates of the mutation rates and the ages of the most recent common ancestor (TMRCA) of the cycad sequences were obtained using BEAST v. 1.4, available from http://beast.bio.ed.ac.uk[101]. We used the GTR+I+G model of nucleotide substitution with estimated base frequencies, gamma shape distribution (with six categories), proportion of invariant sites, and a strict molecular clock with uncorrelated log normal distribution of branch lengths. Posterior estimates of the mutation rate and age of the TMRCA were obtained by Markov chain Monte Carlo (MCMC) analysis, with samples drawn every 500 steps over a total of 25,000,000 steps. The BEAST program was also used to create a Bayesian skyline plot with 10 steps. The analysis was run for 10^7 ^iterations with a burn-in of 10^6 ^under the GTR+I+G model with estimated base frequencies, gamma shape distribution (with six categories), proportion of invariant sites, and a strict molecular clock with uncorrelated log normal distribution of branch lengths. Genealogies and model parameters were sampled every 1000 iterations. All operators were automatically optimized. Convergence of parameters and mixing of chains were followed by visual inspection of parameter trend lines and checking of ESS values by three pre-runs. The effective sampling size (ESS) parameter was found to exceed 100, which suggests acceptable mixing and sufficient sampling. Adequate sampling and convergence to the stationary distribution were checked using TRACER v. 1.3 [102]. Posterior estimates of parameters were all distinctly unimodal (although with wide 95% highest posterior densities), and all parameters were identifiable, despite the relatively low information content in the sequences and the small age range of the sequences.

#### Isolation by migration and estimating of ancestral population size

We used the simulation program IM [1,103] to investigate isolation with a migration model. By applying coalescent simulations and Bayesian computation procedures, IM yielded six demographic parameters, including population-split time, effective population size for the ancestral and two current populations, and migration rates. The posterior probability densities of these parameters are generated by MCMC simulations, and simulations were run with individual simulations being updated 50 million times. Within each simulation, we used the procedure to swap among 10 heated chains (Metropolis coupling) and observed sufficient swapping rate while the simulation was running. These simulations were carried out using several independent runs, with each chain started at a different starting point and initiated with a burn-in period of 100,000 updates. Convergence upon the stationary distribution was assessed by estimating the effective sample size (ESS) for the parameters, based on the autocorrelation of parameter values measured over the course of the run. The analysis was considered to have converged upon a stationary distribution if the independent runs generated similar posterior distributions with a minimum ESS of 100. Each simulation yielded a marginal density histogram for the six parameters of interest. The peaks of the resulting distributions were considered as the maximum likelihood estimates (MLE) of the parameter with credibility intervals equaling the 95% highest posterior density (HPD) intervals.

## Authors' contributions

YCC and KHH carried out the molecular genetic studies and the statistical analysis, and participated in the sequence alignment. TYC, TWH and BAS designed the research and drafted the manuscript. SJM, SH, and XJG took part in material sampling. All authors read and approved the final manuscript.
